# Limitation of therapeutic effort: When less is more

**Published:** 2015-03-30

**Authors:** Catalina Baena Álvarez

**Affiliations:** Specialist in Pain Medicine and Palliative Care. Physical Medicine and Rehabilitation Department. Health Faculty, Universidad del Valle, Cali, Colombia.

The development and proliferation of intensive care units (ICU) and the ability of technology to replace vital functions have added unmeasurable value to medical practice and have changed the way in which culture and science face disease and death. Following progress, new ethical dilemmas arrived: today it is not clear where to draw the line that separates good medical practice and the rational use of technology. Almost always, dignity and life had similar value and, as there were no resources, decisions were based ​​on the principle of dignity, but now resources give more options to sustain life, and in some cases dignity is not under discussion. 

Limitation of therapeutic effort (LTE) is to decide on the status and future of the patient to not apply treatments or therapeutic procedures that will provide little benefit about the suffering or agony the patient is experiencing 1. No starting or withdrawing a therapeutic measure to a patient (with or without capacity to decide), is a decision that challenges the health team in front of multiple scenarios and outcomes where both ethical principles of the effectiveness and the efficacy of each medical action prevail. In these situations, there are two poorly understood terms that can affect the decision: "Limitation" and "obstinacy". In any case they not correspond to a literal interpretation of each word. More than limiting, the therapeutic effort (such as energy) must be transformed in an effort to prolong life, to generate an outcome with the least possible suffering. Similarly, referring to "obstinacy" describes a therapeutic effort as a situation that does not fit into the basic principles of the medical profession 2, even though the intention of that act is surely far from intending to infringe them. 

Deciding not to initiate or discontinue a treatment or intervention gradually becomes part of the medical practice as the end of life comes closer, both in cancer treatment and in the terminal stages of cardiac, neurologic, respiratory or kidney disease; it has been reported that in some studies up to 90% of deaths in ICU were preceded by LTE 3. 

Limitation of therapeutic effort in cancer patients has shown new edges in the XXI century; although cure rates in cancer have also improved, the accessibility to the ICU has modified the admission criteria of patients. In Europe about 15% of ICU admissions correspond to patients diagnosed with cancer, particularly for post-operative (solid tumors) or infectious processes (hematologic neoplasms) 4. Deciding the admission of a cancer patient to the ICU requires an individualized approach, the application of scales appropriate to define a forecast, and the definition of a clear therapeutic target in order to avoid situations surrounding the possible excess of interventions. 

A medical community that accepts death as part of life and understands the relevance of an intervention that is not intended to heal but to provide comfort and convenience (i.e. palliate) probably achieves a better acceptance of terminal illness and, in the same way, prevents the patient and the family of the unnecessary sufferings inherent to life extension when there is no reasonable choice of cure or recovery. 

So, when less is more? When doctors understand that their actions should enhance the dignity of patients, and then make understand the patients who suffer from a disease without curative option (and their families) that there exist medical approaches, scientifically supported and based on evidence, that may help them reach the end of their lives without therapeutic measures or unnecessary interventions, with acceptable symptoms control, and without measures that shorten or lengthen the life beyond its natural outcome. 
